# Development of a novel low‐radiation‐absorbent lok‐bar to reduce X‐ray scattering and absorption in RapidArc^®^ treatment planning and dose delivery

**DOI:** 10.1002/acm2.12066

**Published:** 2017-04-06

**Authors:** Hajime Monzen, Kazuki Kubo, Mikoto Tamura, Masaru Hayakawa, Yasumasa Nishimura

**Affiliations:** ^1^ Department of Medical Physics Graduate School of Medical Science Kindai University 377‐2 Ohno‐Higashi Osaka‐Sayama‐shi Osaka 589‐8511 Japan; ^2^ Business Planning Department Toppan Printing Co. Ltd 1‐5‐1 Taito Taito‐ku Tokyo 110‐8560 Japan; ^3^ Department of Radiation Oncology Faculty of Medicine Kindai University 377‐2 Ohno‐Higashi Osaka‐Sayama‐shi Osaka 589‐8511 Japan

**Keywords:** artifacts, immobilizer, lok‐bar, stereotactic body radiation therapy, volumetric modulated arc therapy

## Abstract

We developed a novel low‐radiation‐absorbent lok‐bar (HM‐bar) that is used to secure the immobilizers to the couch. The aim of this study was to investigate the X‐ray scattering and absorption properties of the HM‐bar in computed tomography (CT) simulation and radiotherapy dose delivery using the Varian Exact™ lok‐bar (VL‐bar) as a benchmark. CT images were obtained with or without lok‐bar, and then each image was visually evaluated for artifacts. The attenuation rates for each lok‐bar were measured using a farmer‐type ionization chamber (PTW30013) and the I'mRT phantom (IBA Dosimetry GmbH). Measurement points were between gantry angles of 110 and 180°. The treatment apparatus was a NovalisTx (Brainlab AG); X‐ray energies were set at 6 MV and 10 MV. In the presence of each lok‐bar, the radiation dose was measured in accordance with 10 volumetric modulated arc therapy–stereotactic body radiation therapy (VMAT‐SBRT) plans for lung cancer. Artifacts were seldom observed in the CT scans of the HM‐bar. The attenuation rate of each lok‐bar was higher when the X‐ray energy was set at 6 MV than at 10 MV. The highest attenuation rate in the VL‐bar was observed at a gantry angle of 112°; the rates were 22.4% at 6 MV and 19.3% at 10 MV. Similarly, the highest attenuation rate for the HM‐bar was also observed at a gantry angle of 112°; the rates were 12.2% and 10.1% at 6 MV and 10 MV, respectively. When the VL‐bar was evaluated, the isocenter dose of the VMAT‐SBRT plans was attenuated by 2.6% as a maximum case. In the case of the HM‐bar, the maximum attenuation was 1.4%. In the measurements of each VMAT‐SBRT plan, the difference of the dose attenuation rate between the VL‐bar and HM‐bar was approximately 1%. The HM‐bar could be used to minimize the occurrence of artifacts and provide good images in CT scans regarding radiotherapy planning and dose calculation. It can be used for patient therapy at hospitals to provide accurate dose delivery because of its low X‐ray scattering and absorption characteristics.

## 1 Introduction

With the development of radiation therapy devices, highly precise radiation therapies such as stereotactic body radiation therapy^1^, ^2^ (SBRT) and volumetric modulated arc therapy (VMAT)^3^ are available in many facilities. To administer these highly precise radiation therapy modalities, a small irradiation field and complex irradiation methods are generally used.^1^, ^2^, ^3^, ^4^ Therefore, in comparison with a conventional therapeutic method, higher precision is required to ensure the accuracy of irradiation positioning, repeatability of patient position and patient fixation precision to control body movement during radiation therapy.^5^ In particular, absorption‐type fixing tools are often used to secure the patient's position in image‐guided radiation therapy.^5^, ^6^, ^7^ The American Association of Physicists in Medicine task group report 176^8^ recommends the use of a couch and various fixtures constructed from carbon‐fiber‐reinforced plastic because of high mechanical strength, low specific density, and relative radio‐translucence.^9^ However, the possible phenomenon of attenuation of the radiation dose should be considered. In a previous study, the couch‐top was modeled and various fixtures were included in the calculation domain^8^ as a countermeasure regarding this concern; it was recommended that the influence of immobilization devices on the absorption and scattering of X‐rays concerning the prescribed dose be considered.

In addition to carrying out the recommended method, positional precision of not only the patient, but also the fixtures and couch are required. The current device used to ensure positional precision of the fixtures and couch is the Varian Exact™ lok‐bar (VL‐bar; MedTec, Orange City, IA, USA); the device is made of metal. Our recently developed low‐radiation‐absorbent lok‐bar (HM‐bar) is designed for better performance relative to the VL‐bar, even if it is present in the irradiation field during radiation therapy for lung, head, neck, and esophageal cancer. In the present study, the usefulness of this HM‐bar and its characteristics were compared with those of the VL‐bar.

## 2 Methods

### 2.A Specification and composition of the HM‐bar

**Figure 1:**
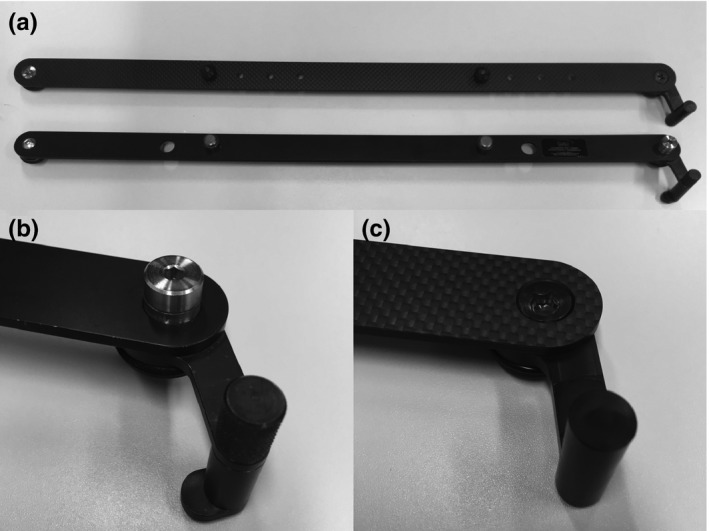
Photographs showing the HM‐bar and the VL‐bar. (a) HM‐bar (upper) and VL‐bar (lower). (b) The component of the VL‐bar that fixes to the couch. (c) The component of the HM‐bar that fixes to the couch. None of the components protrude.

Figure 1 shows the HM‐bar. The HM‐bar has the same security and precision in terms of fixation of the couch compared to the VL‐bar because its specifications were the same as those for the VL‐bar. The main frame of the HM‐bar was constructed from carbon‐fiber‐reinforced plastic boards that were made by hot pressing five layers of epoxy‐resin impregnated sheets; after the carbon board had foamed, it was cut into the correct bar size using a numerical control lathe. The pins were shaped from polyacetal resin blocks using a bench lathe. They were connected using 5 × 30 mm polycarbonate countersunk head screws. The fixing position of the two pins could be controlled by four phases of adjustment. Hence, there was flexibility in selecting the position of fixation of the couch. The only difference regarding the appearance of the HM‐bar was that in contrast to the VL‐bar there was no protrusion of the component used to stabilize the couch (Fig. 1(b) and 1(c)).

### 2.B Computed tomography scan artifacts

The lok‐bars were positioned on the couch and scanned using a 16‐slice computed tomography (CT) scanner (Optima CT580W: GE Healthcare, Milwaukee, WI, USA). The imaging conditions were set with a tube voltage of 120 kV and a 600 mA current. The field of view (FOV) was set at 600 mm based on the shape of the lok‐bars and the I'mRT Phantom (IBA Dosimetry GmbH, Schwarzenbruck, Germany). An acrylic plate was placed on the lok‐bars to secure them to the phantom. Images were obtained without the VL‐bar and HM‐bar, or with the lok‐bars in the same FOV. Subtracted images were prepared after the CT scan; each pixel was subtracted from the CT images without the lok‐bar, or from the CT images with the VL‐bar and HM‐bar, using an Advantage WS workstation (GE Healthcare, Milwaukee, WI, USA). Then, the subtracted images were visually compared for artifacts. In addition, the profile curves were drawn for each subtracted image in the *X*‐axis.

### 2.C Dose calculation using the treatment planning system

The main purpose was to confirm whether dose calculation using the treatment planning system (TPS) would correspond with the measurements concerning the lok‐bars. We assumed that measured attenuation rates were the true values. CT images used for the investigation of the level of artifacts present were prepared for use in the TPS. We drew structures such as each lok‐bar and the I'mRT phantom under the condition that each lok‐bar was included in the dose calculation domain, and the CT scan override was set as “out of function.” The anisotropic analytical algorithm (AAA) and the Acuros XB ver.10 were used as the calculation algorithm. X‐ray energies were set at 6 and 10 MV. The calculation grid was 2.5 mm.

**Figure 2:**
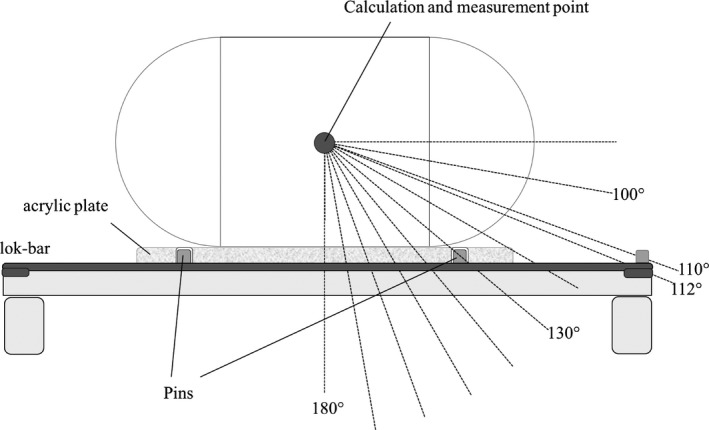
Schematic view of the calculation and measurement geometry.

The dose calculations were as follows. The dose calculation point was set at the center of the phantom. The point was irradiated every 10° at gantry angle between 100 and 180° with 100 monitor unit (MU) at 600 MU/min, and then absorbed dose was calculated. The dose calculation at 112°, which was the stabilizing point for the lok‐bars on the couch, was also examined. Figure 2 shows the schematic view of the calculation geometry. The dose attenuation rate at each angle was calculated using the calculated dose in the presence of the lok‐bar and calculated dose for the same angle in the absence of the lok‐bar. We defined the attenuation rate with recipe (1):

$$Attenuation\;rate=\frac{\left|D_{with\;lok-bar}-D_{without\;lok-bar}\right|}{D_{without\;lok-bar}}.$$

where *D* is the absorbed dose.

### 2.D Dosimeter measurements

To determine the dose attenuation rate for each lok‐bar, a former‐type ionization chamber (PTW30013; PTW, Freiburg, Germany) was installed at the center of the phantom, where the radiation doses were measured at every gantry angle used for the 100‐MU irradiation. All conditions were the same as in the TPS experiment (Fig. 2). The irradiation apparatus was a NovalisTx (Brainlab AG, Munich, Germany) and X‐ray energies were set at 6 and 10 MV. The measured values represented the average of three measurements taken for every angle. The absorbed doses were converted into the dose attenuation rate, as the basis of an absorbed dose measured at every gantry angle in the condition where the couch was without the lok‐bar. The dose attenuation rates were compared with the results for each lok‐bar at every gantry angle and the dose attenuation rates were calculated using the TPS.

Moreover, to study the influence of the lok‐bars on the radiation dose, the experiment was conducted according to the 10 VMAT‐SBRT plans including the irregular and small fields for lung cancer radiation therapy. Each plan was undertaken using an X‐ray energy of 6 MV; the prescription dose was 70 Gy/10 fractions, and two partial round arcs were used (gantry angle, 0–180°). Table 1 shows the summary for 10 VMAT‐SBRT plans.

**Table 1:** Summary of the 10 VMAT‐SBRT plans including PTV volume, field size, and MU per daily prescription dose

Plan number	PTV volume (cm^3^)	Field size (cm)	MU/prescription dose
1	9.4	3.7 × 3.5	1489
2	9.3	3.2 × 3.4	2518
3	39.7	5.6 × 5.0	1973
4	39.2	5.0 × 5.1	1953
5	21.8	4.2 × 5.2	1446
6	44.3	6.7 × 6.8	1926
7	29.3	4.4 × 4.7	1559
8	29.3	4.5 × 5.0	1770
9	139.4	10.1 × 9.9	1643
10	23.5	6.9 × 5.2	1723

## 3 Results

### 3.A Artifacts in the CT scans

**Figure 3:**
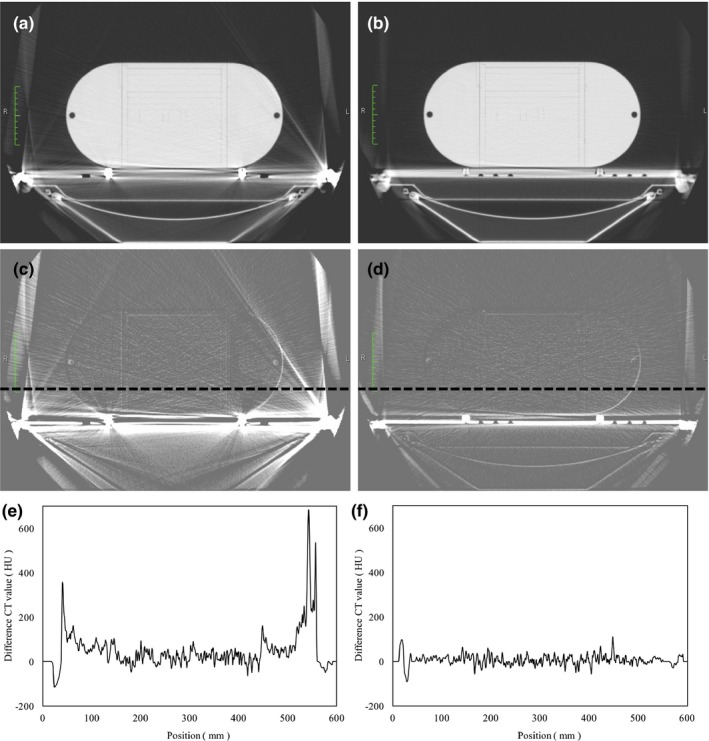
Artifacts produced by the lok‐bars. (a) VL‐bar. (b) HM‐bar. (c) VL‐bar; difference in CT image as a result of the presence of the lok‐bar. (d) HM‐bar; difference in the CT image as a result of the presence of the lok‐bar. (e) Profile curve for the CT values regarding the subtracted image of the VL‐bar (black dotted line) shown in (c). (f) Profile curve for the CT values regarding the subtracted image of the HM‐bar on the *X*‐axis (black dotted line in (d)).

Figure 3 shows the CT images of the VL‐bar and HM‐bar. In the VL‐bar, the metal artifacts from two pins and the component used to stabilize the couch were obvious. However, there were almost no artifacts in the HM‐bar. The maximum difference in the CT values between the couch with and without the VL‐bar was approximately 680 HU as a result of the metal artifacts (Fig. 3(e)). The maximum difference in the CT values between the couch with and without the HM‐bar was approximately 110 HU (Fig. 3(f)). However, the actual difference might be ˂100 HU because it could have been caused by the phantom setup.

### 3.B Dose calculation using the treatment planning system

Table 2 shows the computed attenuation rate regarding irradiation at each gantry angle using the dose calculated by means of the TPS. The X‐ray dose attenuation rate was higher at 6 MV than at 10 MV at all gantry angles, since 6 MV X‐ray has a weak penetrating power compared with that of 10 MV X‐ray. Approximately, 1–2% of the dose attenuation rate was observed when the beam's center axis passed the body section of the lok‐bars (gantry angle, 140–180°). There was no significant difference between the VL‐bar and HM‐bar. The dose attenuation rate of the VL bar showed increase significantly when the beam's center axis passed the pins and the component used to stabilize the couch (i.e., gantry angle 112 and 130°, as shown in Fig. 2) as compared with other angles. The highest dose attenuation rate concerning the VL‐bar was recorded at an angle of 110°. The rates were 16.3% for 6 MV X‐rays and 13% for 10 MV X‐rays when calculated using the AAA. At the same angle, the beam's center axis also passed the protruding part of the VL‐bar on the couch. The dose attenuation rates were 23.7% for 6 MV X‐rays and 19.2% for 10 MV X‐rays when calculated using the Acuros XB.

**Table 2:** Computed radiation dose attenuation rate data for each gantry angle using the treatment planning software

Attenuation rate (%)								
Gantry angle (degree)	VL‐bar				HM‐bar			
	AAA		Acuros XB		AAA		Acuros XB	
	6 MV	10 MV	6 MV	10 MV	6 MV	10 MV	6 MV	10 MV
100	2.1	1.7	2.4	1.8	0.0	0.0	0.0	0.0
110	16.3	13.0	23.7	19.2	0.7	0.6	0.5	0.8
112	10.3	8.4	15.1	12.5	3.0	2.5	4.1	3.5
120	2.8	2.2	2.7	2.3	2.3	1.6	2.5	2.1
130	7.5	5.9	11.4	8.8	3.9	3.1	5.3	4.0
140	1.2	1.0	1.2	1.0	1.6	1.3	2.1	1.6
150	1.4	1.0	1.0	0.8	1.2	1.0	1.8	1.4
160	1.1	0.8	0.9	0.9	0.9	0.8	1.5	1.3
170	1.2	0.7	0.9	0.8	1.0	0.6	1.3	1.2
180	0.9	0.8	0.9	0.7	0.8	0.6	1.4	1.2

For the HM‐bar, the radiation dose attenuation rate was lower than that for the VL‐bar. At a gantry angle of 130°, the calculations were 3.9% for 6 MV X‐rays and 3.1% for 10 MV X‐rays using the AAA. The attenuation rates for each gantry angle were ≤3% except for the gantry angle 130°. The differences in the dose attenuation rate between the HM‐bar and the VL‐bar at maximum differences were approximately 15% for 6 MV X‐rays and 12% for 10 MV X‐rays using the AAA. Similarly, regarding the calculations using the Acuros XB, which gave a much higher value than the AAA, the maximum differences were approximately 22% for 6 MV X‐rays and 18% for 10 MV X‐rays.

### 3.C Dosimeter measurements

**Figure 4:**
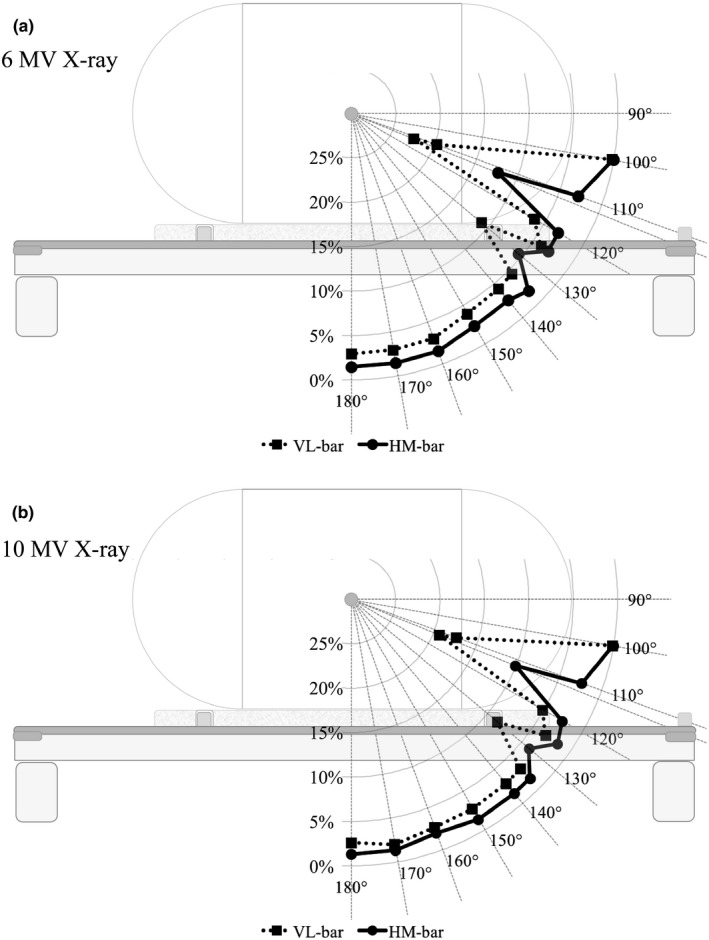
Radiation dose attenuation rate for each lok‐bar. (a) Cases involving 6 MV X‐rays. (b) Cases involving 10 MV X‐rays.

Figure 4 shows the radiation dose attenuation rate for both lok‐bars when measured at all gantry angles. The results show that the dose attenuation rate was higher for 6 MV X‐rays than for 10 MV X‐rays. The highest dose attenuation rate for the VL‐bar was observed at a gantry angle of 112°; the rates were 22.4% for 6 MV X‐rays and 19.3% for 10 MV X‐rays. For the HM‐bar, the highest attenuation rate was also observed at a gantry angle of 112°; the rates were 12.2% for 6 MV X‐rays and 10.1% for 10 MV X‐rays. The highest dose attenuation rate in case of the HM‐bar was approximately 10% lower than that for the VL‐bar. The same trend was also observed at other gantry angles. The highest attenuation rate was observed at a gantry angle of 110°, and the values were 17% for 6 MV X‐rays and 15% for 10 MV X‐rays in the HM‐bar. The protrusion of the component used to stabilize the couch was responsible for these results.

**Figure 5:**
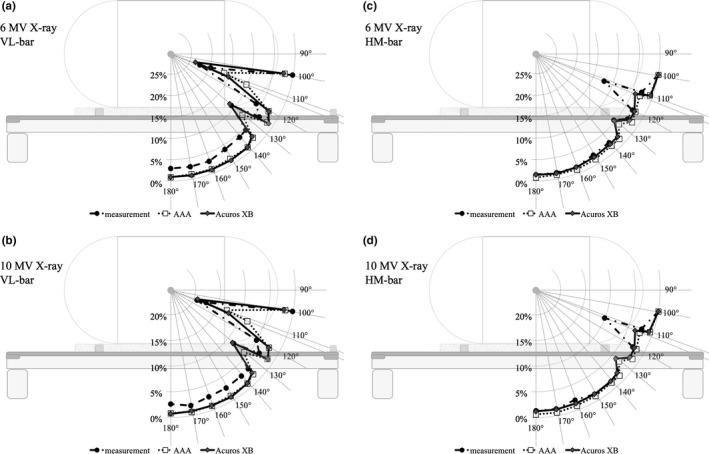
Comparison between the radiation dose attenuation rates calculated using the treatment planning software and the actual measurements using the dosimeter. (a) 6 MV X‐rays; VL‐bar. (b) 10 MV X‐rays; VL‐bar. (c) 6 MV X‐rays; HM‐bar. (d) 10 MV X‐rays; HM‐bar.

The radiation dose attenuation rates were calculated using the TPS and detected by the dosimeter (Fig. 5). For the VL‐bar, the dose attenuation rate for 6 MV X‐rays was underestimated by approximately 2% as compared with the actual measurement at the angle of the beam center when it passed the body of the lok‐bar (gantry angle, 140–180°). When the beam passed the pins and the component of the lok‐bar used to stabilize the couch (gantry angles 112 and 130°), the dose attenuation rate showed good agreement with the calculations produced using the Acuros XB algorithm; however, the AAA algorithm underestimated it by about 4%. The highest dose attenuation rate was detected at an angle of 112° concerning the actual measurement, but the highest dose attenuation rate calculated using the TPS was at 110°. At an X‐ray energy of 10 MV, the results exhibited a similar tendency and the dose attenuation rate was underestimated by approximately 1% relative to the actual measurement at the gantry angle where the beam center passed the body of the lok‐bar (gantry angles, 140–180°). As the beam center passed the pins and the component of the lok‐bar used to stabilize the couch, the dose attenuation rate showed good agreement with the calculations using the Acuros XB algorithm. For the HM‐bar, the difference between the dose attenuation obtained using each calculation algorithm was not significant, and the differences between measurements and calculations agreed within approximately 1%. The highest dose attenuation rate was at a gantry angle of 112° in the actual measurement, but in the calculations, the highest dose attenuation rate was at a gantry angle of 130°. The influence of each lok‐bar on dose attenuation using the VMAT‐SBRT plans for lung cancer is detailed in Table 3. For the VL‐bar, the center dose was attenuated by 2.6% as a maximum case. For the HM‐bar, the maximum dose attenuation was 1.4%. The difference between the VL‐bar and HM‐bar was approximately 1%.

**Table 3:** Influence of two lok‐bars on radiation dose attenuation using the VMAT‐SBRT plans for the treatment of lung cancer

Plan number	Attenuation rate (%)		
	HM‐bar	VL‐bar	Difference
1	1.3	2.4	−1.1
2	1.4	2.6	−1.2
3	0.9	1.8	−0.9
4	1.0	1.9	−0.9
5	0.9	1.8	−0.9
6	0.4	1.0	−0.6
7	1.1	2.3	−1.2
8	1.1	2.0	−0.9
9	1.2	2.1	−0.9
10	0.6	1.3	−0.7
Average	1.0	1.9	−0.9

## 4 Discussion

The HM‐bar is an outstanding tool that has minimal influence on the dose while securing the position of the couch and other fixtures during radiation therapy. The metal in the VL‐bar produces a relatively high number of artifacts during CT scans. However, in the present study, there was no examination of the influences of these metal artifacts on the dose calculation results. There is a possibility that the dose calculation was incorrectly performed. For this reason, the maximum difference in the values calculated by means of CT was considered to be approximately 680 HU when using the VL‐bar (Fig. 3(e)). In other words, it may be concluded that the uncertainty regarding the dose calculation would increase when using the VL‐bar.

Furthermore, the artifacts generated in the CT scan affect the accuracy of the target contours. This problem would become significant when a cone‐beam (CB) CT image contour was photographed during treatment. For example, contouring and dose calculations are generally carried out using CBCT image in adaptive radiotherapy. The CBCT images produced would be inferior in terms of low‐contrast image resolution relative to CT images. It is thought that bigger influences would occur depending on the accuracy of the contour, even if there were few artifacts. In addition, the aggravation of low‐contrast image resolution could also influence image registration involving image‐guided radiation therapy. Therefore, it is important to reduce the occurrence of artifacts and resolve the factors that cause them to appear in CT scans. The HM‐bar generated very few artifacts as compared with the VL‐bar. In other words, the use of the HM‐bar could minimize the problems that affect dose calculation, accuracy of target contour, and precision of image registration in radiation therapy.

The dose attenuation rates for the HM‐bar and VL‐bar were examined in relation to whether or not accurate dose calculation using the TPS is possible. The dose attenuation rate calculated using the TPS and the Acuros XB algorithm was matched with the results obtained using the actual measurements at gantry angles of 110, 112, and 130° in the case of VL‐bar; however, for other gantry angles, it did not match with approximately 4% differences. In the AAA, the gantry angles that showed a high‐dose attenuation rate had been underestimated. This issue could be settled by overriding the contour of the lok‐bars using the appropriate values determined using CT. However, producing a radiotherapy plan to adjust each contour would take a long time and complicate operations.^10^

In this study, it was shown that the angles for the maximum attenuation rate calculated using the TPS differed from those calculated using the actual measurement, even in the case of the HM‐bar. The course of the unmatched gantry angle was not contoured correctly under the influence of artifacts. Moreover, the commissioning work to decide a most suitable CT level is not easy, and it is impossible to take steps to cope with it that depend on the device used for radiation treatment planning. Therefore, the lok‐bars should not be considered in the device used for radiation treatment planning. The best solution for this problem is to use material that had minimal influences on radiation therapy. However, it was impossible to completely prevent radiation dose attenuation using the HM‐bar. In conventional treatment methods, the clinical influences of multiple beam angles are ignored, and the use of a beam traveling along the part of the lok‐bar with a high radiation damping factor is avoided. The institutes do not have the HM‐bar, the results showed that we might need the avoidance angle sections (e.g., 110 ± 3° and 130 ± 3°) in VMAT and SBRT. In the actual radiation doses used in the VMAT‐SBRT plans for lung cancer radiation therapy, we presumed the maximum influence of the lok‐bar on dose attenuation in a clinical setting. The dose attenuation rate could be improved from 2% to 1% by changing from the VL‐bar to the HM‐bar in this study, which could decrease the dose error caused by the lok‐bar. In previous studies, it has been reported that from the theoretical radiobiological point of view, the dose error should be ˂3% of the prescribed radiation dose to maximize the efficacy of the radiation therapy.^11^, ^12^, ^13^ From this stand point, the 1% improvement in dose error when using the HM‐bar is a significant achievement. Furthermore, this 1% difference could be accepted in clinical practice.

## 5 Conclusion

The HM‐bar constructed from low‐radiation‐absorbent material could be used to produce improved CT scan images for radiation therapy planning and dose calculation. This tool will be acceptable in a clinical setting for the improvement of accurate dose delivery.

## Conflict of interest

Hajime Monzen has a consultancy agreement with, and financial interest in, TOPPAN PRINTING CO., LTD, Tokyo.
